# Preoperative abductor muscle strength on the healthy side affects the Timed Up and Go test after total hip arthroplasty in women

**DOI:** 10.1186/s12891-024-08008-6

**Published:** 2024-11-05

**Authors:** Takehiro Kawakami, Takashi Imagama, Yuta Matsuki, Tomoya Okazaki, Takehiro Kaneoka, Kazuhiro Yamazaki, Masaya Ueda, Takashi Sakai

**Affiliations:** Department of Orthopaedic Surgery, Yamaguchi University Graduate School of Medicine, 1-1-1 Minami-Kogushi, Ube, Yamaguchi Prefecture 755-8505 Japan

**Keywords:** Timed up and Go test(TUG), Gluteus medius muscle, Total hip arthroplasty(THA), CT density of muscle

## Abstract

**Background:**

The risk of falls causing periprosthetic fracture has become an issue with the increase in the number of patients undergoing long-term follow-up after total hip arthroplasty (THA) and the aging of patients. The Timed Up and Go test (TUG) is utilized to evaluate fall risk. This study investigated muscle volume around the hip joint based on computed tomography (CT), CT value, and muscle strength to investigate contributing factors to poor TUG 1 year post-THA.

**Methods:**

This study retrospectively investigated 124 patients with unilateral hip osteoarthritis who underwent THA and classified them based on TUG results at 1 year postoperatively into TUG of < 10 s (fast group [103 patients]) and ≥ 10 s (slow group [21 patients]). Body mass index, the volume and CT density of the psoas major and gluteus medius muscles on CT images, pre- and postoperative hip flexion muscle strength, and hip abductor muscle strength were compared in each group.

**Results:**

Age was significantly older and preoperative abductor (fast Group: 1.0 ± 0.3 and slow Group: 0.7 ± 0.3, *P* = 0.003) and flexion muscle strengths (0.9 ± 0.3 and 0.7 ± 0.3, respectively, *P* = 0.02) on the healthy side were significantly lower in the slow group. The gluteus medius muscle demonstrated significantly lower CT density in the slow group on both sides. Nominal logistic regression analysis revealed that age and preoperative healthy abductor muscle strength, which are poor factors for TUG 1 year post-THA, were significantly associated with TUG of ≥ 10 s at 1 year post-THA.

**Conclusions:**

The poor factors for TUG 1 year after THA were age and preoperative abductor muscle strength on the healthy side.

## Introduction

Total hip arthroplasty (THA) in patients with end-stage hip osteoarthritis is considered one of the most successful surgeries in the orthopedic field, and the number of THA is expected to increase as the population ages [1]. The indication for surgery is expanding because the safety of surgery is relatively guaranteed, and the number of elderly people aged 70–90 who undergo THA will increase in the future [2]. Additionally, the 32-year survival rate of implants is good at 80%, and patients after THA have been predicted to continue to get older in the future [3].

Conversely, falls among the elderly have become a serious social problem in recent years [4], and approximately one-third of patients reported a history of falling at least once within the first year post-THA [5]. Falls are associated with periprosthetic hip fractures post-THA. Reportedly, periprosthetic hip fractures are prevalent in elderly people, and treatment is challenging and the long-term mortality rate is high [6, 7]. To prevent periprosthetic hip fractures, the risk of falling after THA needs to be evaluated. Falls may be associated with a decline in lower limb muscle strength and balance during walking [4].

Timed Up and GO test (TUG) is utilized to evaluate fall risk post-THA. TUG is an effective test to screen for dynamic balance disorders related to increased risk of falls [8]. Older age, female sex, and decreased hip flexion strength are adverse factors for TUG [9, 10]. Decreased abduction muscle strength contributes to unstable walking in patients with hip osteoarthritis [11]. The psoas major muscle, which is a hip flexor, helps maintain posture while walking [12]. Nankaku revealed a decrease in preoperative gluteus medius cross-sectional area as a predictor of limping 6 months post-THA in patients with hip osteoarthritis [11]. However, the muscle shape is widely variable, whereas cross-sectional analysis influences the measurement section area, particularly in patients with severe hip deformity. Muscle volume assessment on computed tomography (CT) has been validated in patients with hip osteoarthritis [13]. Although hip function improves after THA surgery, muscle strength on the operated side has been reported to decrease, sometimes lasting more than two years after surgery [14, 15]. Preoperative sarcopenia, a generalized weakness of the muscles, has been reported to result in poor functional outcomes after THA [16]. We believe that measurement of preoperative muscle volume and muscle strength can be a factor in predicting hip function after THA.

Muscle volume around the hip joint based on CT, CT value, and muscle strength were investigated to elucidate contributing factors to poor TUG 1 year after THA.

## Materials and methods

**Fig. 1:**
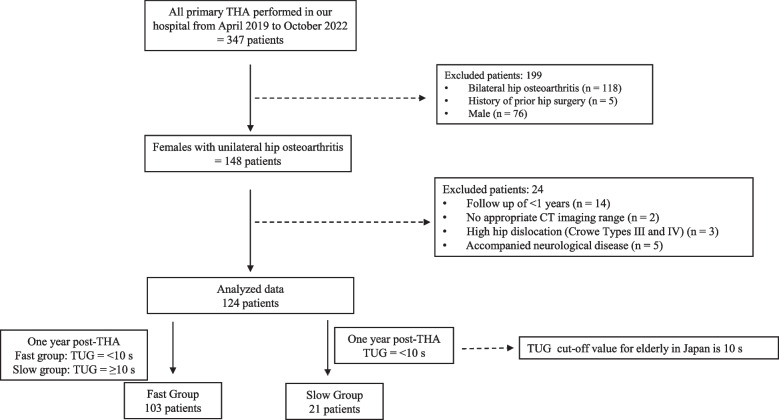
Patient selection flowchart

This retrospective study included 148 female patients with unilateral hip arthritis who underwent primary THA from April 2019 to October 2022 (Fig. 1). All participants were females to avoid the influence of gender differences in muscle mass and skeletal muscle fatty degeneration [17]. This study excluded 14 patients who did not undergo examination 1 year postoperatively, 2 patients with no appropriate CT imaging range, 3 patients with high hip dislocation (Crowe types III and IV in 2 and 1 patients, respectively), and 5 patients with neurological disease. The final participants included 124 patients. The cut-off value for TUG was set at 10 s based on the previous reports [11, 18]. This value is lower than other TUG values recommended for predicting future fall risk, such as TUG 16 s [19] and 13.5 s [20]. However, Arnold et al. suggest that setting TUG to a high value to predict falls may miss many older people at moderate to high risk of falling [18], and we finally set the TUG cut-off value at 10 s.

The patients were categorized into the fast group (< 10 s of TUG, *n* = 103) and the slow group (≥ 10 s of TUG, *n* = 21).

The Ethics Committee and Institutional Review Board of Yamaguchi University Hospital (H2020-068–2) approved the trial protocol.

## Methods

This study evaluated age, height, body mass index (BMI), surgical approach (posterolateral [PL], modified Watson-Jones approach [mWJ], and direct anterior approach[DAA]), femoral components (Mainstay; KYOCERA, Accolade II; Stryker, Exceter; Stryker, Corail; Depuy Synthes, Actis; Depuy Synthes), the volume and CT density of the gluteus medius and psoas muscles assessed with preoperative CT examination, abduction and flexion muscle strength pre-operatively and 1 year postoperatively, postoperative global offset (GO), and history of falls postoperatively.

**Fig. 2:**
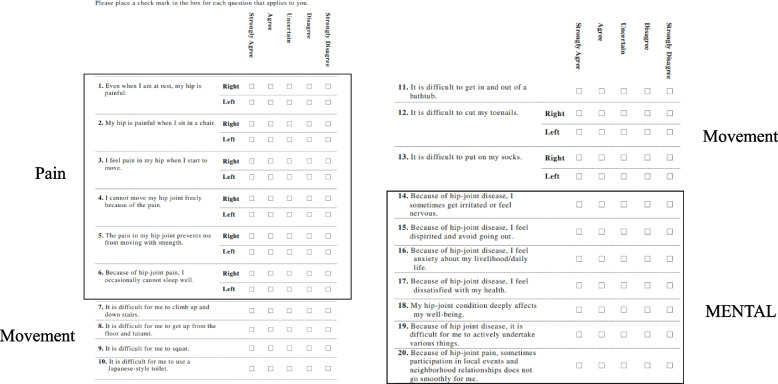
Japanese Orthopedic Association Hip Disease Evaluation Questionnaire (JHEQ). The JHEQ consists of pain (28 points), movement (28 points), and mental (28 points) subscales, with higher scores indicating better outcomes. Questions on the left and right side of the hip joint are to be answered separately, which can be evaluated separately on the affected and healthy side

The same team performed the surgery using an intraoperative CT-based navigation system (CT Hip 1.1, Stryker, Mahwah, NJ). Additionally, we use a CT-based 3-dimensional templating system (ZedHip; LEXI, Tokyo, Japan) for preoperative planning. Moreover, the Japanese Orthopedic Association Hip Disease Evaluation Questionnaire (JHEQ) was used preoperatively and 1 year postoperatively for patient-reported outcome measures [21] The JHEQ consists of pain (28 points), movement (28 points), and mental health (28 points), with higher scores indicating better results. Questions are raised for pain, movement, and mental subscales, with possible scores for each item of 0–4, indicating strongly agree, agree, unsure, disagree, and strongly disagree, respectively) (Fig. 2). The maximum total score is 84 points. Some questions can be answered separately for the left and right sides, thereby differentiating and evaluating the affected from the healthy sides.

### Muscle volume and CT density

CT was performed preoperatively and 2 weeks postoperatively using a helical-roux CT scanner (Aquilion Precision System, Toshiba Medical System, Tokyo, Japan 120 kv, 300 mA). Patients were assessed in the supine position with their pelvis in the neutral position and the lower limb placed in the patient’s natural rotation, wherein the lower leg position is in a relaxed position. We did not enforce lower legs to patella median position because muscle stretch may affect the muscle volumes.

**Fig. 3:**
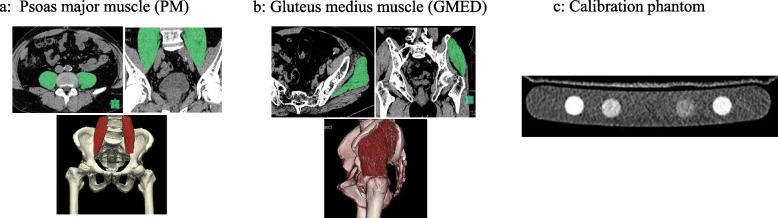
The volume and CT density of the psoas major muscle (PM) and gluteus medius muscle (GMED) were measured using dedicated software. **a** The volume and CT density of the PM were automatically measured using the software application. **b** The GMED was masked every three slices, and the volume and CT density was calculated. **c** A calibration phantom (B-MAS 200: Kyoto Scientific, Japan) was placed in the CT scanner and the CT density was corrected using an equal amount of hydroxyapatite in the bone mineral density reference phantom

Transverse images were captured from the iliac crest to the foot of each patient with a 1-mm slice thickness. Muscle volume and CT density were measured using dedicated software (Synapse Vincent®: Fujifilm Medical Systems, Tokyo, Japan) referring to previous reports (Fig. 3). A dedicated application within the software was used to automatically measure psoas muscle volume and CT density from the lower border of the L3 vertebral body to the suprapubic bone (Fig. 3a). A free-hand draw method was utilized to measure the gluteus medius muscle by tracing the margin of the gluteus medius muscle, referring to previous reports (Fig. 3b) [22]. The gluteus medius muscle volume and CT density were calculated by masking every three slices from the proximal part where the gluteus medius muscle could be seen to the attachment point of the greater trochanter. Artifacts, such as adjacent bones, were removed, and volumes and CT density were calculated. Volume was divided by height squared to correct for volume effects. CT density was adjusted by calculating the equivalent hydroxyapatite amount in muscle area and a calibration phantom (B-MAS 200; Kyoto-Kagaku, Kyoto, Japan) using dedicated software for adjusted HU calculation of the muscle in the CT image based upon reference values measured in bone mineral reference phantom (Fig. 3c). Muscle volume and CT density were measured on the healthy and affected sides, respectively.

### Timed Up and Go test

TUG was measured preoperatively and 1 year postoperatively. The TUG measures the time required by a patient to stand up from an armless chair, walk a distance of 3 m, turn, walk back to the chair, and sit down. The test was performed by walking at their maximum speed.

### Hip abduction and flexion muscle strengths

A handheld dynamometer (ANIMA; uTas F-1) was utilized to measure hip abduction and flexion strength preoperatively and 1 year post-THA. A dynamometer was placed on the outside of the distal thigh to measure hip abduction and flexion muscle strengths in the supine position. The healthy and affected sides were measured thrice times each, and the maximum muscle strength (N) was identified.

### Global offset

**Fig. 4:**
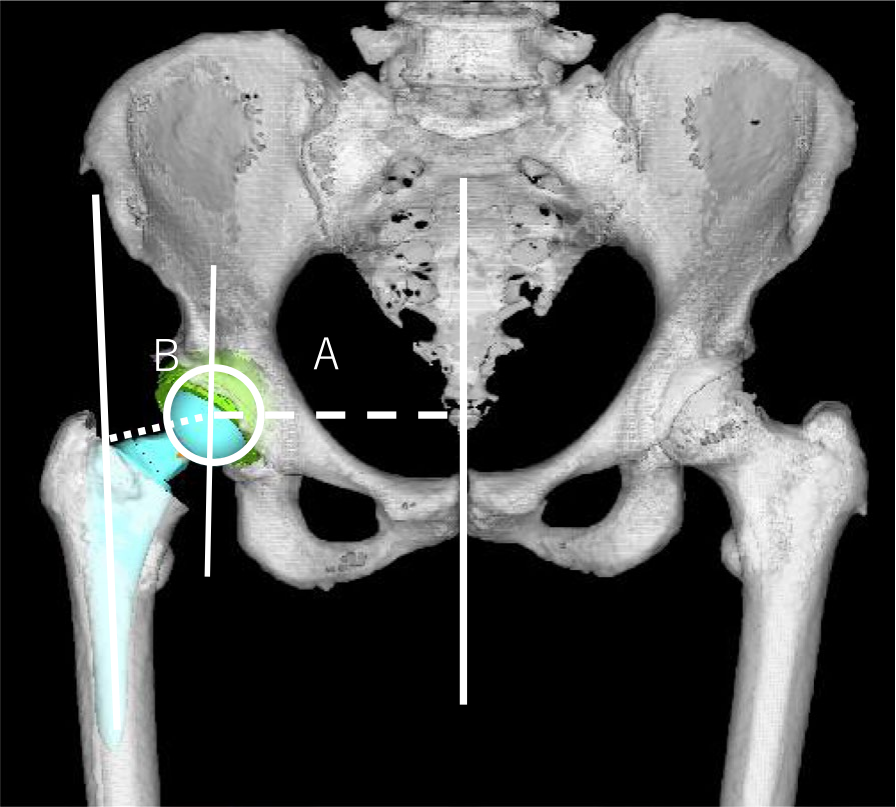
Measurement of offset. **A** Acetabular offset: distance between the cup center and the pubic symphysis. **B** Femoral offset: distance between the cup center and the femoral axis. Global offset are the sum of **A** and **B**

Measurements were conducted using the CT-based three-dimensional templating system (ZedHip; LEXI, Tokyo, Japan) for preoperative planning. Based on previous reports, we transferred preoperative and 2-week postoperative CT images and constructed independent three-dimensional pelvis and femur models, and acetabular and femoral offsets were measured [23]. Acetabular and femoral offsets were measured by projecting onto the functional pelvic plane (Fig. 4). Acetabular offset is the distance between the pubic symphysis and the center of the femoral head, whereas femoral offset is the distance between the center of the femoral head and shaft. Global offset (GO) was the sum of acetabular offset and femoral [23].

### Sample size calculation

Analysis was conducted using G*power (Faul, Erdfelder, Buchner, & Lang, 2009). A power analysis revealed that at least 15 patients in one group and 83 patients in the other were required to compare the groups using a *t*-test, with an effect size of 0.8, a power of 0.8, and an alpha error of 0.05.

### Statistics

This study conducted an analysis using JMP®Pro 15 (SAS Institute Inc). Continuous variables with normal distribution were expressed as mean and standard deviation. The *t*-test and the chi-square test were used for comparisons between groups for quantitative independent data and categorical data, respectively. We determined preoperative factors with a TUG of 10 s 1 year post-THA using univariate analysis and performed nominal logistic analysis. Intraclass correlation coefficients were calculated to assess the intraobserver and interobserver reliability of muscle volume measurements and CT density of the gluteus medius and psoas major muscles. Hence, the author measured the gluteus medius and psoas major muscle volumes and CT density of 30 randomly selected joints twice at an interval of 6 months, and another orthopedic surgeon (MU) evaluated the same patients, and intraobservation reliability and interobserver reliability were assessed.

## Results

The intraobserver and interobserver reliabilities were 0.987 (95% confidence interval [CI]: 0.973–0.994) and 0.893 (95% CI: 0.788–0.948) for the volume measurement (Table 1) and 0.992 (95% CI: 0.983–0.996) and 0.989 (95% CI: 0.976–0.996) for the CT density of the gluteus medius muscle, respectively. The intraobserver and interobserver reliabilities were 0.998 (95% CI: 0.997–0.999) and 0.995 (95% CI: 0.989–0.998) for the volume measurements and 0.951 (95% CI: 0.983–0.996) and 0.978 (95% CI: 0.955–0.990) for the CT density of the psoas major muscle, respectively.

**Table 1:** The intraobserver and interobserver reliabilities of the volume measurement of the gluteus medius and psoas major muscles

	Intraobserver reliability ICC (1,1) (95% CI)	Interobserver reliability ICC (2,1) (95% CI)
***Gluteus medius muscle***		
Volume	0.987 (0.973–0.994)	0.893 (0.788–0.948)
CT density	0.992 (0.983–0.996)	0.989 (0.976–0.996)
***Psoas major muscle***		
Volume	0.998 (0.997–0.999)	0.995 (0.989–0.998)
CT density	0.951 (0.983–0.996)	0.978 (0.955–0.990)

*ICC* Intraclass correlation coefficients, *CI* Confidence interval, *CT* Computed tomography

One year post-THA, 103 (83.1%) patients in the fast group reported a TUG of < 10 s, whereas 21 (16.9%) patients in the slow group exhibited a TUG of ≥ 10 s. Age (fast group: 65.3 ± 10.5 and slow group: 74.4 ± 7.8, *P* < 0.0001) was significantly older in the slow group. Preoperative JHEQ total score on the affected side, preoperative JHEQ movement score and total score on the healthy side, and preoperative JHEQ mental score were significantly lower in the slow group (Table 2). Conversely, there were no significant differences in BMI, femoral components, preoperative JHEQ pain score, preoperative JHEQ movement score on the affected side, as well as pain score on the healthy side.

**Table 2:** Baseline characteristics of Fast and Slow groups

	Fast Group *N* = 103 patients	Slow Group *N* = 21 patients	*P*-value
Age (years)	65.3 ± 10.5 (25–84)^a^	74.4 ± 7.8 (55–86)^a^	< 0.0001
BMI (kg/m^2^)	24.0 ± 5.1 (16.3–40.5)^a^	25.3 ± 5.6 (18.1–39.3)^a^	0.31
**Surgical approach (hips)** **(PL/OCM/DAA)**	62/9/32	9/0/12	0.24
**Femoral components (hips)**			0.17
**Mainstay**	43	9	
**Accolade II**	35	4	
**Corail**	20	5	
**Actis**	3	1	
**Exceter**	2	2	
**TUG (sec)**			
Preoperative	11.2 ± 4.9 (5.7–26.7)^a^	18.3 ± 7.1 (9.2–36.4)^a^	< 0.0001
Postoperative	7.1 ± 1.4 (4.7–9.9)^a^	13.5 ± 5.1 (10.7–35.0)^a^	< 0.0001
**Preoperative JHEQ** **Affect side**			
Pain	6.3 ± 4.9 (0–22)^a^	6.2 ± 4.3 (0–12)^a^	0.90
movement	5.1 ± 5.1 (0–20)^a^	2.2 ± 3.1 (0–8)^a^	0.05
total score	20.4 ± 13.9 (0–68)^a^	13.3 ± 8.3 (0–27)^a^	0.03
**Preoperative JHEQ Healthy side**			
Pain	25.2 ± 5.4 (0–28)^a^	24.3 ± 4.4 (13–28)^a^	0.50
Movement	10.1 ± 4.2 (0–23)^a^	6.1 ± 4.0 (1–14)^a^	0.0002
Total Score	43.9 ± 12.0 (10–75)^a^	35.3 ± 6.5 (26–50)^a^	0.002
**Preoperative JHEQ** Mental	8.7 ± 6.3 (0–28)^a^	4.6 ± 3.3 (0–9)^a^	0.004

*BMI* Body mass index, *PL* Posterolateral, *OCM* Orthopadisehe Chirugie Munchen, *DAA* Direct anterior approach, *TUG* Timed UP and Go test, *JHEQ* Japanese Orthopedic Association Hip Disease Evaluation Questionnaire

^a^mean ± SD (range)

History of falls after THA were significantly more frequent in the slow group (fast group: 10 [9.7%] and slow group: 8 [38.1%] patients, *P* = 0.002).

Preoperative abductor muscle strength (fast group: 1.0 ± 0.3 and slow group: 0.7 ± 0.3, *P* = 0.003) and flexion muscle strength on the healthy side (fast group: 0.9 ± 0.3 and slow group: 0.7 ± 0.3, *P* = 0.02) was significantly lower in the slow group (Table 3). Conversely, no significant difference in abductor or flexion muscle strengths on the affected side was found between the two groups. One year post-THA, abductor and flexion muscle strengths were significantly lower in the slow group on both the affected and healthy sides.

**Table 3:** Preoperative and postoperative hip abductor and flexion muscle strengths

	Fast Group *N* = 103 mean ± SD	Slow Group *N* = 21 mean ± SD	*P*-Value
**Preoperative**			
*** Abductor muscle strength* (Nm/kg)**			
Affected side	0.7 ± 0.3	0.6 ± 0.3	0.05
Healthy side	1.0 ± 0.3	0.7 ± 0.3	0.003
*** Flexion muscle strength* (Nm/kg)**			
Affected side	0.6 ± 0.3	0.6 ± 0.3	0.55
Healthy side	0.9 ± 0.3	0.7 ± 0.3	0.02
**Postoperative(1year)**			
*** Abductor muscle strength* (Nm/kg)**			
Affected side	1.1 ± 0.3	0.8 ± 0.2	< 0.0001
Healthy side	1.2 ± 0.3	0.8 ± 0.3	< 0.0001
*** Flexion muscle strength* (Nm/kg)**			
Affected side	0.9 ± 0.3	0.7 ± 0.3	0.001
Healthy side	1.0 ± 0.3	0.7 ± 0.3	< 0.001

*CT* Computed tomography

The CT density of the gluteus medius was significantly lower in the slow group on both the healthy and affected sides (affected side: fast group: 24.5 ± 13.7 and slow group: 13.4 ± 21.5, *P* = 0.001, healthy side: fast group: 36.6 ± 7.7 and slow group: 27.1 ± 11.5, *P* < 0.0001), and the adjusted CT density of the gluteus medius was significantly lower in the slow group (affected side: fast group: 15.6 ± 12.6 and slow group: 6.2 ± 18.1, *P* = 0.005, healthy side: fast group: 26.3 ± 7.9 and slow group: 19.0 ± 9.7, *P* = 0.0003) (Table 4). No significant difference in the volume of the gluteus medius was found between the affected and healthy sides. No significant differences in volume, density, or adjusted CT density of the psoas major muscle were observed.

**Table 4:** Comparison of volume and adjusted CT density of gluteus medius and psoas major muscles by CT examination

	Fast Group *N* = 103 mean ± SD	Slow Group *N* = 21 mean ± SD	*P*-Value
***Gluteus medius muscle***			
V**olume (ml/m**^**2**^**)**			
Affected side	80.4 ± 16.6	75.7 ± 12.9	0.22
Healthy side	94.7 ± 19.5	88.0 ± 16.4	0.15
**CT density (HU)**			
Affected side	24.5 ± 13.7	13.4 ± 21.5	0.001
Healthy side	36.6 ± 7.7	27.1 ± 11.5	< 0.0001
**Adjusted CT density (mg/cm**^**3**^**)**			
Affected side	15.6 ± 12.6	6.2 ± 18.1	0.005
Healthy side	26.3 ± 7.9	19.0 ± 9.7	0.0003
***Psoas major muscle***			
**Volume (ml/m**^**2**^**)**			
Affected side	21.5 ± 7.0	20.9 ± 5.4	0.72
Healthy side	27.5 ± 7.1	29.0 ± 7.0	0.36
**CT density (HU)**			
Affected side	37.8 ± 7.7	32.5 ± 7.9	0.22
Healthy side	40.7 ± 6.4	37.9 ± 6.9	0.07
**Adjusted CT density (mg/cm**^**3**^**)**			
Affected side	24.5 ± 7.5	23.4 ± 7.6	0.51
Healthy side	29.8 ± 6.2	28.1 ± 6.9	0.26

*CT* Computed tomography

JHEQ was significantly lower in the slow group in terms of movement and total scores on the affected side and movement, total, and mental scores on the healthy side 1 year post-THA (Table 5). No significant differences in pain items were observed between the two groups on both the affected and healthy sides. No significant difference in GO was found between the two groups on both the affected and healthy sides (Table 6).

**Table 5:** The results of postoperative JHEQ between the fast and slow groups

			Fast Group *N* = 103 mean ± SD	Slow Group *N* = 21 mean ± SD	*P*-Value
Postoperative JHEQ	Affected side	Pain	25.1 ± 4.3	24.7 ± 5.8	0.72
		Movement	19.3 ± 6.7	15.8 ± 8.0	0.04
		Total Score	68.2 ± 13.5	60.8 ± 17.0	0.03
	Healthy side	Pain	26.3 ± 3.5	26.3 ± 4.6	0.98
		Movement	20.5 ± 5.8	16.7 ± 7.8	0.01
		Total Score	70.6 ± 12.0	64.7 ± 15.2	0.05
	Mental		23.7 ± 5.3	20.3 ± 7.0	0.01

*JHEQ* Japanese Orthopedic Association Hip Disease Evaluation Questionnaire

**Table 6:** The results of Acetabular offset, Femoral offset and Global offset between the fast and slow groups

		Fast Group *N* = 103 mean ± SD	Slow Group *N* = 21 mean ± SD	*P*-Value
Acetabular offset (mm)	Affected side	86.8 ± 4.7	87.5 ± 4.2	0.58
	Healthy side	88.3 ± 5.6	87.4 ± 4.0	0.56
Femoral offset (mm)	Affected side	33.4 ± 7.2	37.0 ± 6.4	0.06
	Healthy side	32.8 ± 6.4	35.3 ± 5.9	0.14
Global offset (mm)	Affected side	120.2 ± 8.1	124.5 ± 7.2	0.06
	Healthy side	121.1 ± 7.9	122.8 ± 7.3	0.43

Univariate analysis revealed age (*P* < 0.0001), abductor (*P* = 0.003) and flexor muscle strengths on the healthy side (*P* = 0.02), and the CT density of the gluteus medius muscle on the affected (*P* = 0.001) and healthy sides (*P* < 0.0001) as preoperative significant factors for TUG of ≥ 10 s. The factors that caused TUG to be ≥ 10 s in 1 year post-THA were investigated using nominal logistic regression analysis, revealing age (odds ratio [OR]: 1.13, 95% CI: 1.0–1.2, *P* = 0.0002) and preoperative abductor muscle strength on the healthy side (OR: 0.07, 95% CI: 0.0007–0.7, *P* = 0.02) as significant factors (Table 7). TUG at one year after THA correlated with preoperative abductor muscle strength on the healthy side (*R* = -0.27 *P* = 0.002). TUG at one year after THA correlated with age (*R* = 0.36 *P* < 0.001).

**Table 7:** Factors that caused TUG to be ≥ 10 s in 1 year post-THA investigated using nominal logistic regression analysis

	β	Standardized β	95%CI	*P*-value
Age	0.12	0.39	0.07–0.17	< 0.0001
Abductor muscle strength in the healthy side (Nm/kg)	− 2.4	− 0.25	0.004 –4.0	0.004

## Discussion

A most important result of this study was that preoperative abductor muscle strength on the healthy side and age were associated with poor TUG results 1 year post-THA using the nominal logistic regression analysis. The slow group demonstrated significantly lower preoperative abductor muscle strength on the affected and healthy sides, preoperative flexion strength on the healthy side, preoperative CT density of the gluteus medius muscle on the affected and healthy sides, and age than the fast group. The results indicate that strength and muscle quality of the healthy side, but not the affected side, as well as the gluteus medius muscles are important for TUG results 1 year post-THA, contrary to the hypothesis of this study.

The CT density is useful for assessing muscle quality [13]. Goodpaster et al. revealed a high correlation between CT values and skeletal muscle steatosis, which decreases by 1 HU when the lipid concentration increases by 1 g/100 ml [24]. Additionally, increased intramuscular adipose tissue has reduced muscle performance and metabolic status [24]. Reportedly, a large burden is placed on the healthy knee joint in patients with unilateral hip osteoarthritis when walking [25, 26]. Hence, the affected side first experiences muscle weakness and a decreased sense of balance, and the function of the healthy side declines as the affected side worsens [27]. In particular, fatty degeneration of the gluteus medius muscle on the healthy side develops and abductor muscle strength is reduced even with unilateral hip osteoarthritis, as the disease progresses. Ohmori et al. [23] revealed that preoperative one-leg standing time on the healthy side of patients with unilateral hip osteoarthritis was associated with 10-m walking speed 1 year post-THA, confirming the results of the present study. The slow group exhibited lower CT density and preoperative abductor muscle strength in the healthy gluteus medius muscles than the fast group. Adjusted CT densities were obtained, with similar results, as CT density can be biased by the environment. Preoperative abductor muscle strength was a significant factor in the nominal logistic analysis. Therefore, early intervention in patients with unilateral hip osteoarthritis is recommended before the function of the gluteus medius muscle on the healthy side deteriorates.

The results of this study showed that TUG was poor 1 year after THA if there was preoperative reduction in abductor muscle strength on the healthy side. Although pre- and post-operative pain scores were not significantly different between the two groups, the slow group had lower pre-operative and post-operative movement scores and lower mental scores.

TUG can be conducted in a short time and is considered useful for evaluating fall risk [28]. Additionally, the Centers for Disease Control and Prevention recommends TUG as a screening test for fall risk in older adults [29]. The history of falls post-THA was significantly frequent in the slow group. Additionally, TUG is useful for predicting postoperative functional outcomes in patients with hip prostheses and is a predominant preoperative and postoperative evaluation [29–32]. Conversely, literature investigating factors that cause poor TUG after THA is limited, and this study is the first to report that preoperative abductor muscle strength on the healthy side is a factor that causes poor TUG 1 year post-THA. Previous studies have revealed that a decrease in GO on the operated side reduces abductor muscle strength and declines walking ability [33]. This study revealed no significant difference between the two groups because a CT-based three-dimensional templating system was used for preoperative planning. The offset was planned to be no > 10 mm compared to the healthy side; thus, the effect of the offset was considered minor.

This study has limitations. This study did not evaluate CT 1 year postoperatively due to radiation exposure. However, previous reports indicate that the gluteus medius muscle muscle persists even 2 years post-THA on the affected and healthy sides [15]. Additionally, this study only evaluated the psoas major and gluteus medius muscles. Evaluation of other muscles is desirable for a practical understanding of functional impairment in hip osteoarthritis because hip movements require complex coordination of multiple muscles. In the future, our volumetric/qualitative CT analysis will be useful for assessing other muscles and providing better information compared to traditional analyses based on muscle cross-sectional area.

## Conclusion

We measured preoperative muscle volume and CT density of the psoas major muscle and gluteus medius muscle, evaluated the longitudinal change of these muscle strengths, and investigated whether they were factors for poor TUG 1 year post-THA. The unfavorable factors for TUG 1 year post-THA include preoperative abduction strength in the healthy side and age. Surgical treatment is recommended before fatty degeneration and weakness of gluteus medius muscles develop in patients with unilateral hip arthritis.

## Data Availability

The datasets used and analysed during the current study available from the corresponding author on reasonable request.
